# Jellyfish Collagen Hydrolysate Alleviates Inflammation and Oxidative Stress and Improves Gut Microbe Composition in High-Fat Diet-Fed Mice

**DOI:** 10.1155/2022/5628702

**Published:** 2022-08-08

**Authors:** Zhe Lv, Chongyang Zhang, Wei Song, Qingsong Chen, Yaohui Wang

**Affiliations:** ^1^Department of Emergency, The First Hospital of Qinhuangdao, Qinhuangdao, China 066000; ^2^Ministry of Natural Resources (MNR) Key Laboratory of Marine Eco-Environmental Science and Technology, First Institute of Oceanography, Ministry of Natural Resources, Qingdao, China 266061; ^3^Laboratory of Marine Ecology and Environmental Science, Qingdao National Laboratory for Marine Science and Technology, Qingdao, China 266235

## Abstract

The collagen from jellyfish has many beneficial effects, including antioxidant, anti-inflammatory and immune-modulatory activities. However, whether jellyfish collagen hydrolysate (JCH) has any effects on high-fat diet-induced obesity remains unknown. Consequently, we in the present study orally administrated JCH in high-fat diet-fed mice to explore its effects on body weight gain, inflammatory and oxidative status, and cecum microbe composition. The results showed that oral administration of JCH prevented the body weight gain in high-fat diet-treated mice. Meanwhile, glucose, triglycerides, and total cholesterol level in serum were maintained by JCH administration. Furthermore, JCH administration alleviated oxidative stress by increasing the GSH content and decreasing the level reactive oxygen species in the liver and improved inflammatory response by decreasing the expression of TNF-*α*, IL-1*β*, and IL-8 gene in the liver and ileum. Importantly, JCH administration helps recover the alteration of microbiota composition induced by high-fat diet, and the genus *Romboutsia* may critically involve in the beneficial effects of JCH administration. In conclusion, our results indicated that JCH could be potentially used for the prevention and treatment of diet-induced obesity.

## 1. Introduction

Obesity has become a worldwide problem for decades, and its prevalence causes growing threat to public health. Obese individuals are accompanied by high incidence of chronic diseases, including metabolic syndrome and cancers. Many popular therapies for the treatment of obesity including diet pills are found to have side effects when they are taken for a long period [1]. Consequently, there is an urgent need to explore safe and side-effect free methods for preventing the development of obesity.

Natural agents which have health-promoting effects are promising antiobesity substances and arouse increasing attention in recent years. The jellyfish had been used as a tasty food from ancient China. Recently, the nutritional value of jellyfish has been recognized which makes it popular in other countries. The jellyfish is rich in collagenous protein with no crude fat [2]. The collagen from jellyfish has been proved to exert many beneficial effects, including antioxidant, anti-inflammatory, and immune-modulatory activities and lipid-lowering effect [3–6]. Since obesity is always accompanied by overaccumulation of reactive oxygen species (ROS) and overproduction of inflammatory cytokines [7], these effects of jellyfish collagen suggested that it could be potentially used for preventing and treating obesity. However, there is no report related to the beneficial role of jellyfish collagen on high-fat diet-induced obesity until now.

Dietary fat intake has been proved to the major factor which induces obesity. High-fat diet-induced obesity in rodents has been long used as an appropriate model for the study of dietary obesity [8]. Consequently, we explored the effects of jellyfish collagen hydrolysate on high-fat-induced body weight gain in mice. Meanwhile, the effects of jellyfish collagen hydrolysate on inflammatory and oxidative status in high-fat diet-fed mice were also explored. Finally, whether jellyfish collagen hydrolysate administration could alter gut microbe composition was studied.

## 2. Materials and Methods

### 2.1. Preparation of Jellyfish Collagen Hydrolysate (JCH)

Jellyfish (*Nemopilema nomurai*) was washed with distilled water and treated with 0.1 mol/L sodium hydroxide at 4°C for 48 h to remove noncollagenous proteins as previously described [2]. The insoluble fraction was resuspended in sodium phosphate buffer and hydrolyzed with 2% protamex at 50°C for 8 h. The resulting solution was heated at 100°C for 12 min and then centrifuged at 8000 g at 4°C for 8 min. Finally, the supernatant were freeze-dried and stored at -20°C for use.

### 2.2. Animal Experiment

Male C57BL/6J mice (7 weeks old) were provided by the SLAC Laboratory Animal Central (Shanghai, China). All animals were randomly divided into three groups: mice fed on a low-fat (10 kcal% fat) diet were designated as the control group (Control, *n* = 8), mice fed on a high-fat (45 kcal% fat) diet were designated as the high-fat group (Highfat, *n* = 8), and mice fed on a high-fat diet and orally gavaged with JCH (50 mg/kg body weight once a day) were designated as the JCH administration group (Jellyco, *n* = 8). All diets were provided by Research Diets (Guangzhou, China). The experiment lasted 2 months, and all animals had free access to feed and water. During the treatment, body weight was recorded, and body weight gain was calculated. The experimental protocol was approved by the Protocol Management and Review Committee of the First Institute of Oceanography of China.

### 2.3. Sample Collection

At the end of the experiment, blood sample was collected from the retroorbital sinus and then centrifuged at 3000 g for 12 min to obtain serum. Liver and ileum tissues were immediately snap-frozen in liquid nitrogen for further analysis. Cecum content were collected and stored at -80°C for the analysis of microbiota composition.

### 2.4. Determination of Serum Biochemical Indicators and Inflammatory Cytokines

The concentration of serum glucose, triglycerides (TG), total cholesterol (TC), and glutathione (GSH), as well as inflammatory cytokines (TNF-*α*, IL-1*β*, and IL-8), was determined by using commercial kits (BYabscience).

### 2.5. RT-qPCR Analysis

Total RNA was extracted from liver and ileum tissues by using the TRIzol Reagents (Invitrogen) and then was reverse-transcribed into cDNA by using the RevertAid RT Kit (Thermo Scientific). Primers used for RT-qPCR were as follows: TNF-*α*, forward 5′-CCCACGTCGTAGCAAACCAC-3′, reverse 5′-GCAGCCTTGTCCCTTGAAGA-3′; IL-1*β*, forward 5′-TGCCACCTTTTGACAGTGATG-3′, reverse 5′-AAGGTCCACGGGAAAGACAC-3′; IL-8, forward 5′-TGCATGGACAGTCATCCACC-3′, reverse 5′-ATGACAGACCACAGAACGGC-3′. Then, qPCR was performed with a total volume of 10 *μ*L assay solution containing 1 *μ*L cDNA, 0.4 *μ*L forward primer, 0.4 *μ*L reverse primer, 3 *μ*L deionized water, 0.2 *μ*L ROX, and 5 *μ*L SYBR Green Mix (Applied Biosystems) as previously described [9].

### 2.6. Determination of GSH Content and GPX Activity in the Liver and Ileum

The concentration of GSH and GSSG, as well as the activity of GPX in the liver and ileum tissue, was measured by using commercial kits (BYabscience).

### 2.7. Determination of ROS Level in the Liver

The level of ROS in the liver was determined as previously described [10]. Briefly, fresh liver tissue was embedded in a freezing medium (Sakura) and snap-frozen in methylbutane solution (Sigma) at -80°C. Then, 10 *μ*m sections were stained with dihydroethidium (Sigma-Aldrich) for 25 min at 37°C. Representative pictures were collected under a fluorescence microscopy (Leica). Relative abundance of ROS fluorescence was determined using Image Browser software (Leica).

### 2.8. Gut Microbe Profiling

DNA from cecum samples was extracted, and the bacterial 16S rDNA gene (V3 + V4 regions) was amplified with specific primers (forward, 5′-ACGGRAGGCWGCAGT-3′; reverse, 5′-TACCAGGGTATCTAATCCT-3′) by performing PCR reactions. The PCR products were purified, and sequencing libraries were generated and analyzed as previously described [11]. Operational taxonomic units were used to analyze with the RDP classifier algorithm. QIIME was performed for the analysis of *α*- and *β*-diversity and principal coordinate analysis.

### 2.9. Statistical Analysis

All data were analyzed using a one-way ANOVA followed by Student-Newman-Keuls post hoc tests using SPSS 19.0. All data were expressed as means ± SEM, and the results were determined to be significant at *P* < 0.05.

## 3. Results

### 3.1. Jellyfish Collagen Hydrolysate Decreased Body Weight Gain and Improved Serum Biochemical Indicators and Inflammatory Cytokine Concentration in High-Fat Diet-Fed Mice

**Figure 1:**
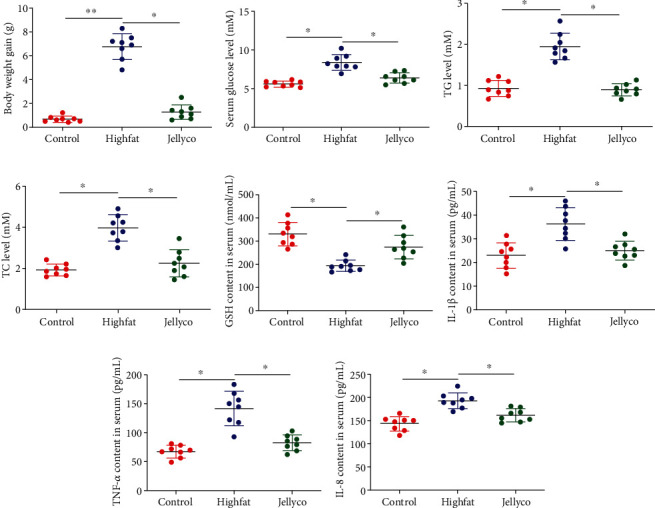
Jellyfish collagen hydrolysate decreased body weight gain and improved serum biochemical indicators and inflammatory cytokine concentration in high-fat diet-fed mice: (a) body weight gain, (b) glucose level, (c) TG level, (d) TC level, (e) GSH content, (f) IL-1*β* content, (g) TNF-*α* content, and (h) IL-8 content. Data are expressed as means ± SEM, *n* = 8. ^∗^*P* < 0.05, ^∗∗^*P* < 0.01.

As shown in Figure 1, mice in the Highfat group had significantly higher body weight gain and higher level of glucose, TG, and TC in serum when compared with mice in the Control group, whereas no significant changes in these parameters were observed between mice in the Control and Jellyco group. Mice in the Highfat group had significantly lower GSH content and higher content of IL-1*β*, TNF-*α*, and IL-8 in serum when compared with mice in the Control group, whereas no significant changes in the abovementioned parameters were observed between mice in the Control and Jellyco group.

### 3.2. Jellyfish Collagen Hydrolysate Alleviated Inflammation and Oxidative Stress in Liver of High-Fat Diet-Fed Mice

**Figure 2:**
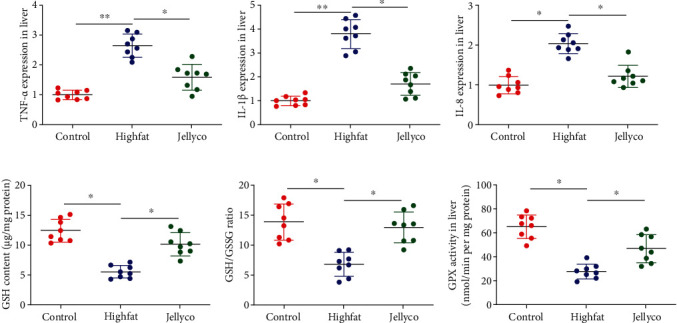
Jellyfish collagen hydrolysate alleviated inflammation and oxidative stress in liver of high-fat diet-fed mice. Gene expression of TNF-*α* (a), IL-1*β* (b), and IL-8 (c), (d) GSH content, (e) GSH/GSSG ratio, and (f) GPX activity. Data are expressed as means ± SEM, *n* = 8. ^∗^*P* < 0.05, ^∗∗^*P* < 0.01.

As shown in Figure 2, the hepatic gene expression of IL-1*β*, TNF-*α*, and IL-8 were significantly increased in mice in the Highfat group than those in the Control group, whereas their expression was significantly decreased in mice in the Jellyco group than those in the Highfat group. Meanwhile, GSH content, the ratio of GSH to GSSG, and GPX activity were significantly decreased in mice in the Highfat group than those in the Control group, whereas these parameters were significantly increased in mice in the Jellyco group than those in the Highfat group.

### 3.3. Jellyfish Collagen Hydrolysate Prevented Hepatic ROS Accumulation in High-Fat Diet-Fed Mice

**Figure 3:**
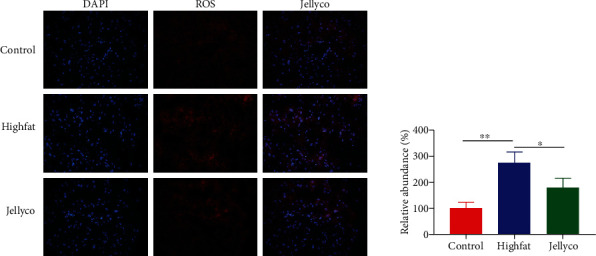
Jellyfish collagen hydrolysate prevented hepatic ROS accumulation in high-fat diet-fed mice. Data are expressed as means ± SEM, *n* = 3. ^∗^*P* < 0.05, ^∗∗^*P* < 0.01.

As shown in Figure 3, high-fat-diet resulted in the overaccumulation of ROS in the liver of mice, whereas jellyfish collagen hydrolysate administration significantly decreased hepatic ROS content in mice.

### 3.4. Jellyfish Collagen Hydrolysate Alleviated Inflammation and Oxidative Stress in Ileum of High-Fat Diet-Fed Mice

**Figure 4:**
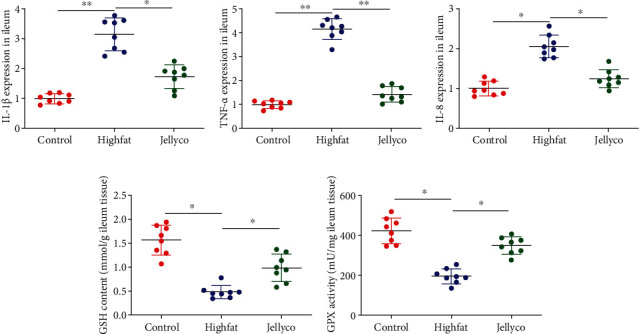
Jellyfish collagen hydrolysate alleviated inflammation and oxidative stress in ileum of high-fat diet-fed mice. Gene expression of IL-1*β* (a), TNF-*α* (b), and IL-8 (c), (d) GSH content, and (e) GPX activity. Data are expressed as means ± SEM, *n* = 8. ^∗^*P* < 0.05, ^∗∗^*P* < 0.01.

As shown in Figure 4, the gene expression of IL-1*β*, TNF-*α*, and IL-8 in ileum was significantly increased in mice in the Highfat group than those in the Control group, whereas their expression was significantly decreased in mice in the Jellyco group than those in the Highfat group. GSH content and GPX activity were significantly decreased in mice in the Highfat group than those in the Control group, whereas these parameters were significantly increased in mice in the Jellyco group than those in the Highfat group.

### 3.5. Jellyfish Collagen Hydrolysate Altered Cecum Microbe Profiling in High-Fat Diet-Fed Mice

**Figure 5:**
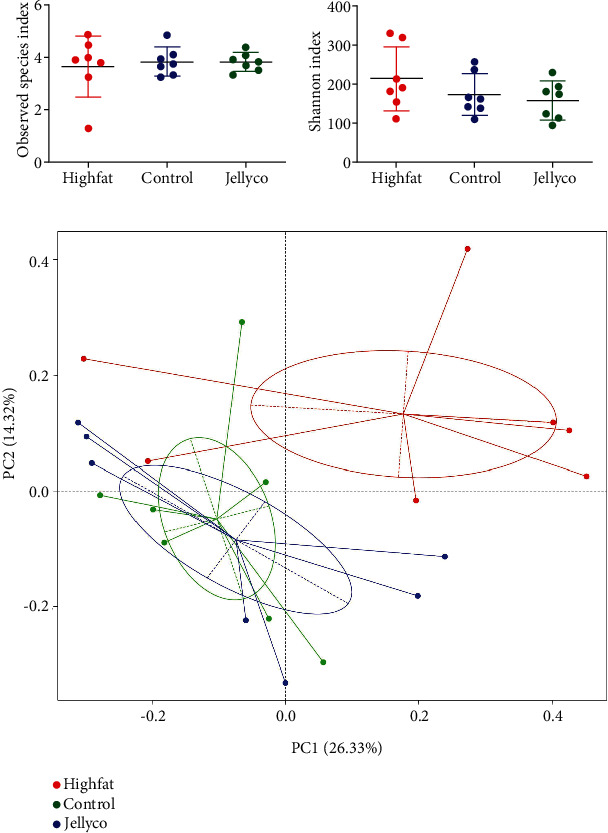
Effects of jellyfish collagen hydrolysate on *α*- and *β*-diversity in cecal microbiota in high-fat diet-fed mice: (a) observed species index, (b) Shannon index, and (c) PCoA analysis based on unweighted UniFrac distance. Data are expressed as means ± SEM, *n* = 7.

No significant difference was observed in *α*-diversity as indicated by observed species (Figure 5(a)) and Shannon index (Figure 5(b)) among the three treatment groups. The *β*-diversity as indicated by PCoA based on unweighted UniFrac distance indicated that the overall microbial structure in mice in the Highfat group was clearly separated away from those in mice in the Control and Jellyco group, whereas the microbial structure in mice in the Control group was not separated away from that in mice in the Jellyco group (Figure 5(c)).

**Figure 6:**
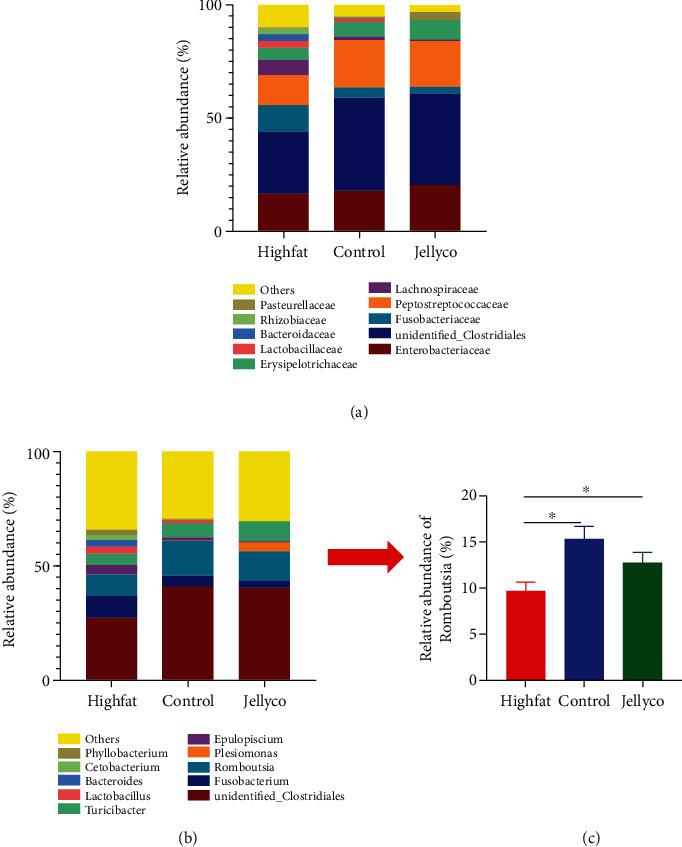
Effects of jellyfish collagen hydrolysate on relative abundance of the cecum microbial species in high-fat diet-fed mice. (a)Relative abundance of bacteria classified at a family-level taxonomy. (b) Relative abundance of bacteria classified at a genus-level taxonomy. (c) Relative abundance of *Romboutsia*. Data are expressed as means ± SEM, *n* = 7. ^∗^*P* < 0.05.

Enterobacteriaceae, Clostridiales, Fusobacteriaceae, and Peptostreptococcaceae were the main microbes at the family level (Figure 6(a)). Mice in the Highfat group had lower Clostridiales abundance and higher Fusobacteriaceae abundance when compared with those in the Control and Jellyco groups, whereas microbe abundance in the family level in mice in the Control group was similar to those in mice in the Jellyco group. *Fusobacterium*, *Romboutsia*, *Clostridiales*, *Plesiomonas*, and *Epulopiscium* were the main microbes in the genus level (Figure 6(b)). Notably, the relative abundance of *Romboutsia* was significantly lower in mice in the Highfat group than those in the Control and Jellyco groups (Figure 6(c)).

## 4. Discussion

With great biocompatibility and penetrability, collagen and its peptides could be used as bioactive substances. Aquatic animals including jellyfish are excellent source of collagen [12]. Jellyfish collagen was proved to be harmless and exerts widely biological effects on human cells [3]. In the present study, we found that oral administration of JCH prevented the body weight gain in high-fat diet-treated mice. Meanwhile, glucose, TG, and TC level in serum were maintained by JCH administration. Furthermore, JCH administration alleviated oxidative stress and inflammatory response which are often accompanied with obesity. Notably, JCH administration helps recover the alteration of microbiota composition induced by high-fat diet.

Jellyfish is enriched in collagenous protein, which has abundant hydrophobic amino acids. These amino acids have better emulsifying ability and possess strong antioxidant ability [6, 13]. Therefore, JCH was supposed to have higher antioxidant ability than other protein sources. The antioxidant effects of JCH have been studied both *in vitro* and in *vivo*. Jellyfish collagen peptide, as well as crude protein and protein fractions, showed strong hydroxyl radical- and superoxide anion-scavenging activities *in vitro* [14, 15]. The *in vivo* antioxidant effects of JCH were proved as serum GPX activity, and hepatic SOD activity was increased in aging mice after administration with JCH [2]. In the present study, we found that GSH content, which is the most vital intracellular antioxidant, was significantly increased both in the liver and ileum when the high-fat diet-treated mice were administrated with JCH. Moreover, ROS level in the liver was decreased after JCH administration. These results broaden our knowledge on the antioxidant effects of JCH in different experimental models.

Jellyfish collagen can activate innate and acquired immune response. Specifically, jellyfish collagen enhanced inflammatory cytokine secretion through activating TLR4 and NF-*κ*B signaling pathways [4]. Importantly, collagenase inhibited the immune-stimulation effects of the extract from jellyfish on human cells, which indicated that collagen is the active substance [16]. However, an *in vivo* study showed that both JC and JCH improved immunity in mice [17]. We further found that JCH administration decreased inflammatory cytokine contents in serum and their expression in both liver and ileum in high-fat diet-induced mice. These results confirmed that JCH had strong anti-inflammation effects; although, the related mechanism needs to be further elucidated.

The intestinal microbiota modulate host energy homeostasis and can be alter by dietary structure such as high-fat diet. Consumption of high-fat diet often led to decreased Bacteroidetes abundance and increased Firmicutes abundance [18]. Additionally, the intestinal microbe diversity was usually decreased in high-fat diet-fed mice [19]. JCH-administrated mice and control mice had the similar microbiota composition which was different from these of high-fat diet-fed mice. As we known, this is the first study indicating the beneficial effects of JCH on the modulation of gut microbes. Notably, the abundance of *Romboutsia* was decreased by high-fat diet treatment which is in line with previous study [20], whereas its abundance was recovered by JCH administration. The genus *Romboutsia* maintains health of the host and exerts many metabolic capabilities including carbohydrate utilization, metabolic end products, and fermentation of single amino acids [21]. These results suggested that the genus *Romboutsia* may be the major target of JCH. However, how the microbes were involved in the modulation effects of JCH and whether *Romboutsia* plays critical roles remain to be elucidated in the future works.

In conclusion, our results suggested that JCH administration protected mice from high-fat diet-induced obesity and hyperglycemia and hyperlipidemia. Meanwhile, JCH administration helps maintain oxidative and inflammatory status in the liver and intestine of high-fat diet-fed mice. Importantly, JCH administration retrieved cecum microbe composition, and the genus *Romboutsia* may critically involve in the beneficial effects of JCH. Our results indicated that JCH could be potentially used for the prevention and treatment of obesity.

## Data Availability

The data used to support the findings of this study are available from the corresponding author upon request.
